# Pulmonary tuberculosis diagnostic delays in Chad: a multicenter, hospital-based survey in Ndjamena and Moundou

**DOI:** 10.1186/1471-2458-12-513

**Published:** 2012-07-09

**Authors:** Ndeindo Ndeikoundam Ngangro, Doudeadoum Ngarhounoum, Mosurel N Ngangro, Ngakoutou Rangar, Mahinda G Siriwardana, Virginie Halley des Fontaines, Pierre Chauvin

**Affiliations:** 1 Inserm, UMRS, 707, Paris, France; 2 Université Pierre et Marie Curie-Paris6, UMRS, 707, Paris, France; 3 Hôpital Régional, Moundou, Chad; 4 Ministère de la santé publique, Direction générale des activités sanitaires, Ndjamena, Chad; 5 Hôpital général de référence, Ndjamena, Chad; 6 Faculté des sciences de la santé, Université de Ndjamena, Ndjamena, Chad

**Keywords:** Tuberculosis, Delay, Diagnosis, Treatment

## Abstract

**Background:**

Tuberculosis remains one of the leading causes of morbidity and mortality in low-resource countries. One contagious patient can infect 10 to 20 contacts in these settings. Delays in diagnosing TB therefore contribute to the spread of the disease and sustain the epidemic.

**Objectives:**

The aim of this study was to assess delays in diagnosing tuberculosis and the factors associated with these delays in the public hospitals in Moundou and Ndjamena, Chad.

**Methods:**

A structured questionnaire was administered to 286 new tuberculosis patients to evaluate patient delay (time from the onset of symptoms to the first formal or informal care), health-care system delay (time from the first health care to tuberculosis treatment) and total delay (sum of the patient and system delays). Logistic regression was used to identify risk factors associated with long diagnostic delays (defined as greater than the median).

**Results and discussion:**

The median [interquartile range] patient delay, system delay and total delay were 15 [7–30], 36 [19–65] and 57.5 [33–95] days, respectively. Low economic status (aOR [adjusted odds ratio] =2.38 [1.08-5.25]), not being referred to a health service (aOR = 1.75 [1.02- 3.02]) and a secondary level education (aOR = 0.33 [0.12-0.92]) were associated with a long patient delay. Risk factors for a long system delay were a low level of education (aOR = 4.71 [1.34-16.51]) and the belief that traditional medicine and informal care can cure TB (aOR = 5.46 [2.37-12.60]).

**Conclusion:**

Targeted strengthening of the health-care system, including improving patient access, addressing deficiencies in health-related human resources, and improving laboratory networks and linkages as well as community mobilization will make for better outcomes in tuberculosis diagnosis.

## Background

Tuberculosis (TB) is one of the leading causes of morbidity and mortality: 9.2 million new cases of TB and 1.7 million deaths due to this disease were reported worldwide in 2007. The majority of these cases occurred in developing countries, particularly in Asia and Africa [1]. In limited-resource countries, one contagious patient can infect 10 to 20 people during the natural history of the disease [2]. Lin X et al. found that 30 days of infectious disease is enough for the bacillus to pass from the index case to the household members [3]. Consequently, any delay in the diagnosis, care and treatment of TB patients not only exposes them to severe morbidity and a greater risk of mortality, but it also contributes to the spread of the epidemic [4-7]. Thus, one of the main goals of TB control programs should be the prompt diagnosis and treatment of TB patients.

TB is one of Chad’s major public health concerns [8]. In 2009, the prevalence of TB was 480/100,000 population, with an annual incidence estimated at 283/100,000 population and a specific mortality of 63/100,000 population [8]. The disease has been the target of a national control program since 1990, and the DOTS strategy was adopted in 1994. TB care and treatment are free in Chad. Patients with symptoms suggestive of TB are identified when they visit a first-level health service and are subsequently referred to a hospital, where a diagnosis of TB can be confirmed. The main diagnostic tools used are the sputum smear test and chest radiography. When the diagnosis is confirmed, standard treatment regimens are prescribed in accordance with World Health Organization (WHO) recommendations.

A study conducted at a hospital in Ndjamena in 2003 determined the TB diagnostic delay to be 75 days. However, the authors did not clearly distinguish between the patient delay and the health-care system delay [9]. The objectives of our study were to investigate pulmonary TB diagnostic delays and to identify factors associated with these delays in order to strengthen the TB prevention program. For the period from the onset of symptoms to the initiation of TB treatment, we sought to distinguish the “patient delay” (time to the first access to care, whether formal or informal) and the “health-care system delay” (time from the first access to care to the initiation of TB treatment).

## Methods

### Setting

A multicenter questionnaire-type survey was conducted from August to October 2009 in three hospitals, two of which are in the Chadian capital, which has the largest number of TB patients (the Hôpital Général de Référence de Ndjaména [**HGRN]** and the Hôpital de l’Union [**HU]**). Both serve mainly local and urban TB patients. The third hospital, Hôpital de Moundou (**HM)**, is the regional hospital for the Western Logone region (440 km south of Ndjamena)**.** Regular hospitals are designed to serve a population of 100,000 to 200,000, but referral hospitals have a population base larger than this. The population of Ndjamena is 833,531, and of the 650,000 inhabitants of Western Logone, 142,000 live in Moundou. Patients are supposed to visit a health center first. From there, under the referral system, the more severe cases are sent to district hospitals, then to regional hospitals and, lastly, to the HGRN.

### Study population

Newly diagnosed cases of pulmonary TB aged 15 years or older were recruited consecutively and prospectively. The TB cases were classified according to the guidelines of the Chadian TB control program (WHO guidelines). Patients with other lung diseases or extrapulmonary TB, those who declined to give their consent and those who were too weak to answer the questionnaire were excluded from this study. Assuming a frequency of extended total delay of 60% among individuals exposed to a risk factor and of 40% among those not exposed, the study required a sample size of least 225 patients.

### Data

A semi-structured questionnaire was used to collect the data. It was translated into Arabic and Sara when necessary. The questionnaires were filled out by trained interviewers who conducted face-to-face interviews shortly after diagnosis. The patients’ medical records were cross-checked to confirm and complete the data.

The outcome variables were the patient delay (PD; defined as the time interval between the onset of a cough lasting more than 15 days and/or of major symptoms according to the national TB control program guidelines, i.e., night sweats, weight loss, fever and respiratory symptoms− all the cases were reviewed by a pneumologist to date the onset of TB symptoms − and the first formal or informal health care received); the health-care system delay (HSD; defined as the time interval between the previously mentioned care and the initiation of TB treatment); and the total delay (TD; defined as the sum of the patient and system delays). The delays were estimated in number of days. Delays were considered extended when they were longer than their respective median values.

The independent variables to be studied were chosen after an intensive literature review. They were the individual’s demographic and socioeconomic characteristics, such as gender, age (divided into five groups), rural residency, defined as living outside the city (yes/no), health insurance status (yes/no), and level of education (in five groups of increasing numbers of years of education). Economic status was assessed by calculating a wealth score based on housing status, the construction quality of the dwelling, the sources of drinking water and electricity, the type of sanitation, the ownership of certain items (such as a car, a motorbike, a bicycle, a refrigerator or a television) and the case’s occupational status. We also asked the patients how they would pay the additional expenses. The answers were grouped into five categories: the household’s savings, a loan, financial help from relatives or friends, selling his/her belongings, and earnings from continuing to work. We also asked the patients if one of their friends or relatives was a health-care worker (yes/no).

Three medical findings were considered as well: the presence of hemoptysis (yes/no), the result of the smear test (positive or negative) and the patient’s HIV serological status (positive, negative, unknown).

Knowledge and attitudes concerning TB were assessed with questions regarding the cause of TB, its mode of transmission, its treatment, the link between TB and AIDS, and the primary care received.

Distance between the patient’s residence and the closest health facility was divided into three categories (≤ 1 km, between 1 and 5 km, and ≥ 5 km). Lastly, whether or not the case had been referred to the hospital by a primary care facility was examined.

### Statistical analysis

The distributions of the independent variables with the three different delays were compared using a chi-square test (or Fischer’s exact test where the numbers were small), and quantitative variables were compared using the (non-parametric) Wilcoxon test and the Kruskal and Wallis test. The associations between the ordinal variables (age and wealth score) and the outcomes of interest were assessed for trends. Next, since the delays differed according to the three hospitals, we performed bivariate analysis to make the same comparisons after adjusting for the study site and examined whether there were any interactions. Lastly, we included all the variables with a *p*-value ≤ 0.20 in bivariate analysis and selected them by backward analysis, fitting a logistic regression model for each delay separately. In multivariate analysis, the categories for knowledge of TB treatment were medical care, no response and other responses. The categories for the first health care received were formal (health center, hospital, pharmacist or private doctor) and informal (other responses), and the means for paying the additional expenses were classified according to the ability (savings, work) or inability (other responses) to pay. Epidata 3.1 software was used to build the database. Statistical analyses were performed with SAS 9.2.

### Ethical issues

Since there is no ethics committee in Chad, research authorization was obtained from the Chadian Health Ministry. Each patient had been informed of the study’s objectives and his/her right to decline to participate. Verbal informed consent was obtained before every interview. No act that could harm the patients’ dignity or physical integrity was committed during this study.

## Results

### Population characteristics

**Figure 1:**
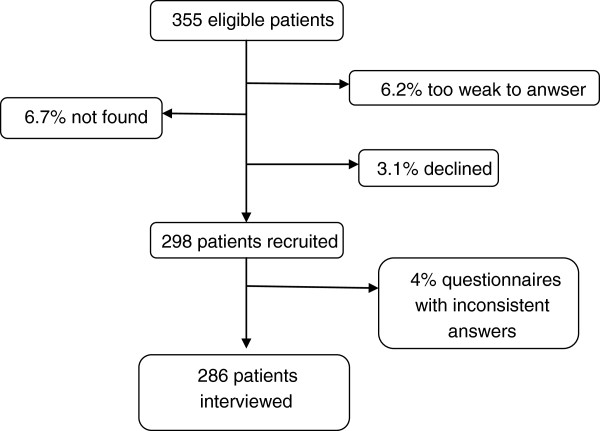
Study recruitment.

Two-hundred and eighty-six newly diagnosed patients were included in the analysis (Figure 1). They were mainly men (67.1%). The median age was 32 years, with less than a fourth of this population being over the age of 41. The education level was low: one-fifth of the population had no education, and only one-tenth of the patients had reached a postgraduate level. Only a minority (17.5%) of the patients lived in a rural area. The average size of the patients' households was 6.1 persons. Half of them were unemployed and had no income. More than 80% of the smear tests were positive. One-fifth of the patients were HIV-positive, and one-third of them had not been tested for HIV. Very few patients (13%) had health insurance, and more than half of them (60.4%) expected financial help from their relatives. One-third of the patients sought treatment by visiting a hospital, 22% by buying drugs on the informal market, 21% by visiting a health center, 13% by using traditional medicine, less than 8% by consulting a private doctor, and 3.5% by consulting a pharmacist. Only 2.1% of them did not seek health care.

### Comparison of the hospital populations

The patients at the HM and the HGRN seemed to be older than those at the HU (p≤0.001), and the patients at the HU were likely to be more educated (p < 0.0001) (Table 1). The wealth scores were higher for the HM and the HGRN than for the HU (p = 0.02). Unemployment also seemed to be more frequent for the HU than for the other two facilities (p < 0.01). There was a higher rate of HIV-positive serology for the HGRN (29.4%) than for the HM (20.3%) and the HU (5.8%), and the HIV serological status of more than half of the patients was unknown at the HU and the HM compared to only one-fifth at the HGRN (p < 0.0001). Because the HSD (p < 0.0001) and TD (p = 0.0002) were much longer for the HGRN, bivariate analysis was adjusted for the hospital.

**Table 1:** Characteristics of the study hospitals

	**Study population**	**Hôpital de l’Union**	**Hôpital Général de Ndjamena**	**Hôpital de Moundou**	***p***
	**Median [IQ]**	**Median [IQ]**	**Median [IQ]**	**Median [IQ]**	
Median patient delay (days)	15 [7-30]	14 [7-21]	15 [7-30]	15 [10–23.5]	0.30
Median health-care system delay (days)	36 [19–65]	35 [20–70]	45 [23–67]	22 [11-40]	0.0001
Median total delay (days)	57.5 [33–95]	56 [32–93]	68 [41–101]	40 [27–63]	0.0002
Median age (years)	32 [26-41]	28 [23-35]	35 [28–45]	32 [27-40]	0.001
Median wealth score	13 [10-18]	14 [10-17]	14 [10-19]	12 [10–14.5]	0.02
Median number of years of education	6 [4-10]	10 [6-10]	6 [4-10]	4 [0–6]	0.0001

***IQ:****Interquartile range.*

### Risk factors associated with an extended patient delay

Once adjusted for the study hospital (Table 2), protective factors were a higher level of education, having health insurance, the belief that people hide their TB, having a health professional among one’s relatives, and the primary care having been obtained by consulting a pharmacist. On the other hand, an extended PD was associated with a remote community health facility, selling one’s belongings in order to pay the additional expenses, and not knowing how TB is transmitted. In multivariate analysis (Table 3), an extended PD was associated with a low wealth score, an intermediate education level, misconceptions about TB treatment, and having no referral to a hospital.

**Table 2:** Factors associated with delays exceeding their median value (univariate analysis )

	**Size**	**Median PD [IQ]**	**Percentage of patients ≥ the median PD**	***P***	**Median HSD [IQ]**	**Percentage of patient ≥ the median HSD**	***P***	**Median TD [IQ]**	**Perecentage of patients ≥ the median TD**	***P***
***Sociodemographic characteristics***										
**Gender**										
Male	192	15.0 [9.0 – 30. 0]	58.3	0.03	35.0 [18.5-66.0]	48.4	0.31	61.5 [33.0 – 94.0]	52.6	0.26
Female	94	11.5 [7.0 – 30.0]	44.7		39.5 [21.0-65.0]	55.3		52.0 [34.0 -89.0]	44.7	
**Age groups (years)**										
15 to 24	60	14.0 [7.0 – 25.5]	48.3	0.42	45.0 [20.0 – 70.0]	58.3	0.08	65.5 [33.0 – 95.0]	56.7	0.31
25 to 34	103	15.0 [7.0 - 30.0]	50.5		31.0 [19.0 – 54.0]	40.8		49.0 [31.0 – 88.0]	42.7	
35 to 44	67	15.0 [7.0 – 27.0]	55.2		44.0 [16.0 – 64.0]	55.2		57.0 [33.0 – 88.0]	49.3	
45 to 55	37	15.0 [10.0 – 30.0]	62.2		35.0 [21.0 – 78.0]	48.7		63.0 [40.0 – 108.0]	54	
55 and over	19	21.0 [10.0 – 60.0]	68.42		46.0 [20.0 – 65.0]	68.4		67.0 [45.0 – 109.0]	63.2	
**Wealth score**										
1^st^ quartile (lowest)	78	15.0 [10.0 – 30.0]	61.5	0.10	49.0 [23.0 – 68.0]	60.3	0.26	71.0 [42.0 -115.0]	60.3	0.19
2^nd^	68	15.5 [7.0 – 30.0]	60.3		33.0 [18.0 – 59.0]	48.5		55.5 [37.5 – 88.0]	48.5	
3^rd^	79	14.0 [7.0 – 24.0]	48.1		33.0 [18.0 – 60.0]	45.6		51.0 [30.0 – 90.0]	45.6	
4^th^ (highest)	61	14.0 [7.0 – 30.0]	44.3		35.0 [20.0 – 66.0]	47.5		48.0 [28.0 -88.0]	44.3	
**Numbers of years of education**										
0	58	17.0 [10.0 – 31.0]	66.7	0.005	42.0 [21.0 – 68.0]	51.9	0.07	64.5 [35.0 – 115.0]	55.6	0.42
1 to 4	43	15.0 [10.0 – 30.0]	67.4		42.0 [18.0 – 66.0]	60.5		61.0 [36.0 – 110.0]	53.5	
5 to 6	77	15.0 [7.0 - 30.0]	52		45.0 [23.0 – 69.0]	55.8		64.0 [40.0 – 90.0]	54.6	
7 to 10	87	13.0 [7.0 – 21.0]	39.1		35.0 [19.0 – 65.0]	48.3		50.0 [30.0 – 85.0]	44.8	
≥ 10	24	21.0 [12.0 – 30.0]	62.5		22.5 [14.5 – 39.0]	25		46.5 [31.5 -92.5]	37.5	
**Health insurance**										
Yes	39	12.0 [7.0 – 30. 0]	35.9	0.02	26.0 [12.0 – 63.0 ]	35.9	0.06	37.0 [25.0 – 86.0]	33.3	0.04
No	247	15.0 [7.0 -30.0]	56.7		40.0 [20.0 – 66.0]	53		61.0 [36.0 – 95.0]	52.6	
**How the patients planned to pay the additional expenses**										
Savings	51	14.0 [7.0 – 22.0]	71.4	0.01	38.0 [17.0 – 55.0]	57.1	0.38	56.0 [33.0 – 80.0]	71.4	0.8
Loan	7	30.0 [7.0 – 45.0]	45.1		43.0 [21.0 – 59.0]	51		73.0 [48.0 – 105.0]	49	
Help from relatives	167	15.0 [7.0 – 30.0]	50.9		41.0 [19.0 – 67.0]	55.1		58.0 [33.0 – 95.0]	50.3	
Working	28	15.0 [7.0 – 40.0]	60.7		35.0 [21.0 – 67.0]	35.7		54.5 [38.5 – 85.0]	46.4	
Selling belongings	25	30.0 [17.0 -45.0]	84		29.0 [23.0 – 61.0]	44		68.0 [47.0 – 115.0]	56	
**Rural residence**										
Yes	50	20.5 [10.0 – 45.0]	64	0.12	54.5 [23.0 – 100.0]	66	0.01	93.0 [48.0 – 123.0]	66	0.02
No	236	15.0 [7.0 – 30.0]	51.7		34.5 [19.0 – 60.0]	47.5		53.5 [32.5 – 88.0]	46.6	
***Clinical aspects***										
**Hemoptysis**										
Yes	61	19.0 [10.0 – 30.0]	60.7	0.25	54.0 [26.0-72.0]	59	0.15	75.0 [39.0 – 113.0]	60.7	0.08
No	225	15.0 [7.0- 30.0]	52		35.0 [18.0 – 60.0]	48.4		55.0 [32.0 – 88.0]	47.1	
**HIV serological status**										
Negative	118	14.0 [7.0 -30.0]	46.5	0.13	44.0 [21.0 – 71.0]	56.8	0.06	62.5 [36.0 – 105.0]	53.4	0.04
Positive	62	16.0 [9.0 -30.0]	62.9		41.5 [21.0 - 64.0]	54.8		66.5 [45.0 – 99.0]	59.7	
Unknown	106	15.0 [7.0 – 30.0]	55.7		31.5 [14.0 – 60.0]	41.5		49.5 [30.0 – 83.0]	40.6	
**Do people hide their TB?**										
Yes	224	14.0 [7.0 -30.0]	49.6	0.02	41.0 [20.0 – 66.0]	52.7	0.25	61.5 [33.0 – 92.0]	52.7	0.10
No	46	20.0 [10.0 – 30.0]	71.7		33.5 [19.0 – 66.0]	47.8		51.0 [37.0 – 108.0]	45.7	
Did not know	16	18.5 [10.0 – 25.5]	62.5		17.5 [10.0 – 47.5]	31.3		46.5 [27.5 – 63.5]	25	
***Knowledge, attitudes and beliefs***										
**Knew what causes TB**										
Yes	37	14.0 [7.0 – 30.0]	46	0.38	32.0 [17.0 71.0]	40.5	0.22	52.0 [36.0 – 99.0]	46	0.72
No	249	15.0 [7.0 – 30.0]	55		38.0 [20.0 – 64.0]	52.2		58.0 [33.0 – 93.0]	50.6	
**Knew how TB is transmitted**										
Yes	58	9.5[7.0 – 21.0]	34.5	0.001	35 [20-65]	48.3	0.77	49.0 [30.0 – 78.0]	44.8	0.46
No	228	15.0[8.5 – 30.0]	58.8		36.5 [19-65.5]	51.3		59.5 [34.5 – 94.0]	51.3	
**Knew that TB treatment was free**										
Yes	111	15.0 [7.0 – 25.0]	55	0.80	23.0 [12.0 – 51.0]	35.1	0.0003	45.0 [28.0 – 75.0]	37.8	0.002
No	175	15.0 [7.0- 30.0]	53.1		46.0 [25.0 – 69.0]	60.6		67.0 [39.0 -101.0]	57.7	
**Is there a link between AIDS and TB?**										
Yes	102	14.0 [7.0 – 30.0]	47.1	0.18	35.0 [19.0 – 70.0]	49	0.04	52.5 [30.0 – 93.0]	49	0.04
No	98	15.0 [7.0 – 30.0]	60.20		44.5 [24.0 – 66.0]	60.2		69.5 [48.0 – 108.0]	59.2	
Did not know	86	15.0 [10.0 – 21.0]	54.7		28.0 [15.0 – 51.0]	41.9		47.0 [31.0 – 73.0]	40.7	
**What treatment can cure TB?**										
Self- medication	5	15.0 [15.0 – 20.0]	80	0.02	12.0 [10.0 – 30.0]	20	0.0001	30.0 [25.0 – 32.0]	20	0.0001
Medical care	120	10.0 [7.0 – 21.0]	40.8		25.5 [17.0 – 52.0]	37.5		45.0 [30.5 – 74.5]	35.8	
No answer	114	18.0 [10.0 – 30.0]	63.2		38.0 [19.0 – 70.0]	53.5		63.5 [37.0 – 105.0]	55.3	
Nothing	4	22.5 [11.0 – 30.0]	75		50.5 [27.0 – 63.0]	75		73.0 [38.0 -93.0]	50	
Traditional medicine	43	15.0 [11.0 – 30.0]	60.5		57.0 [43.0 – 74.0]	81.4		75.0 [58.0 – 107.0]	79.1	
*Access to and use of health services*										
**Referral by a health facility**										
Yes	127	14.0 [7.0 – 30.0]	46.5	0.03	35.0 [19.0 – 64.0]	49.6	0.81	62.0 [35.0 – 91.0]	52	0.63
No	159	15.0 [8.0 – 30.0]	59.8		36.0 [19.0 – 66.0]	51.6		56.0 [30.0 -95.0]	48.4	
**Distance from home to the closest service**										
≤ 1km	146	14.0 [7.0 - 24.0]	48	0.01	35.0 [19.0 -65.0]	49.3	0.08	52.0 [34.0 -89.0]	46.6	0.14
1 to 5 km	109	15.0 [7.0 - 30.0]	55		35.0 [19.0 -61.0]	47.7		58.0 [30.0 – 90.0]	50.5	
≥ 5 km	30	30.0 [30.0 - 45.0]	80		55.0 [26.0 – 71.0]	70		89.0 [52.0 -115.0]	66.7	
**First health care received**										
Self-medication	6	18 [14 – 35]	66.7	0.14	38.5 [33 – 43]	50	0.006	56.0 [45.0 – 75.0]	50	0.04
Health center	62	15.0 [7.0 – 30.0]	61.3		39.5 [19.0 – 66.0]	51.6		57.5 [33.0 – 93.0]	50	
Hospital	78	15.0 [10.0 – 30.0]	61.5		20.5 [10.0 – 53.0]	32		40.5 [27.5 – 75.0]	38.5	
Pharmacist	10	9.0 [7.0 – 14.0]	20		34.5 [19.0 -49.0]	40		43.0 [37.0 – 56.0]	20	
Private doctor	23	14.0 [7.0 – 21.0]	39.1		35.0 [20.0 – 85.0]	47.8		61.0 [30.0 – 103.0]	52.2	
No health care	6	30.0 [15.0 – 30.0]	83.3		48.0 [20.0 – 71.0]	50		63.5 [49.0 – 101.0]	50	
Informal drug market	63	14.0 [7.0 – 30.0]	47.6		44.0 [23.0 – 69.0]	63.5		64.0 [37.0 -113.0]	54	
Traditional medicine	38	14.0 [7.0 – 30.0]	47.4		54.0[34.0 – 81.0]	71		68.5 [50.0 – 110.0]	73.7	
**Knew a health professional**										
Yes	101	14.0 [7.0 – 27.0]	42.6	0.006	43.0 [20.0 – 66.0]	56.4	0.17	63.0 [36.0 – 95.0]	55.5	0.22
No	185	15.0 [9.0 – 30.0]	60		34.0 [19.0 – 65.0]	47.6		56.0 [32.0 – 93.0]	47	
**Hospital**										
**HU**	69	14.0 [7.0 – 21.0]	44.9	0.06	35.0 [20.0 -70.0]	49.3	0.0002	56 [32-93]	46.4	0.0001
**HGRN**	153	15.0 [7.0 – 30.0]	52.9		45.0 [23.0 -67.0]	60.1		68 [41-101]	60.8	
**HM**	64	15.0 [10.0- 23.5]	65.5		22.0 [11.0 – 40.0]	29.7		40 [27-63]	28.1	

***IQ:****Interquartile range.*

**Table 3:** Factors associated with delays exceeding their median value (bivariate analysis, adjusted for the hospital)

	**Extended patient delay OR [95% CI]**	**P**	**Extended health system delay OR [95% CI]**	**P**	**Extended total delay OR [95% CI]**	**P**
***Sociodemographic characteristics***						
**Gender**						
Male	1		1		1	
Female	0.60 [0.36-1.00]	0.46	1.31 [0.79-2.19]	0.30	0.70 [0.42-1.17]	0.17
**Age groups (years)**						
15 to 24	1		1		1	
25 to 34	0.99 [0.52-1.89]		0.51 [0.26- 1]		0.58 [0.30-1.13]	
35 to 44	1.18 [0.58-2.42]		0.91 [0.44-1.88]		0.73 [0.35-1.52]	
45 to 55	1.63 [0.70-3.82]		0.63 [0.27-1.48]		0.81 [0.34-1.91]	
55 and over	2.06 [0.67-6.32]	0.52	1.44 [0.45- 4.54]	0.13	1.13 [0.37-3.47]	0.49
**Wealth score**						
1^st^ quartile (lowest)	2.00 [1.01-4.00]		**2.13 [1.05- 4.33]**		**2.57 [1.25-5.26]**	
2^nd^	1.73 [0.85-3.56]		1.49 [0.72-3.1]		1.79 [0.85-3.75]	
3^rd^	1.21 [0.61-2.39]		1.09 [0.54-2.18]		1.30 [0.64-2.62]	
4^th^ (highest)	1	0.17	1	0.12	1	0.06
**Numbers of years of education**						
0	1.07 [0.39- 2.96]		**4.29 [1.42-13.02]**		2.71 [0.96-7.67]	
1 to 4	1.12 [0.39-3.23]		**6.07 [1.91-19.26]**		2.36 [0.81-6.91]	
5 to 6	0.61 [0.24-1.58]		**4.42 [1.55-12.62]**		2.27 [0.86-5.96]	
7 to 10	**0.39 [0.15- 0.99]**		**2.87 [1.03-7.98]**		1.38 [0.54-3.54]	
≥ 10	1	0.02	1	0.03	1	0.19
**Health insurance**						
No	1		1		1	
Yes	**0.41 [0.20- 0.85]**	0.01	0.54 [ 0.26- 1.12]	0.10	0.5 [0.24-1.05]	0.06
**How the patients planned to pay the additional expenses**						
Savings	1	0.02	1	0.21	1	0.51
Loan	3.34 [0.59-18.86]		1.18 [ 0.24-5.82]		2.43 [ 0.43- 13.78]	
Help from relatives	1.29 [0.66-2.48]		1.58 [0.81-3.08]		1.48 [0.76- 2.90]	
Working	2.50 [0.90-6.75]		0.61 [0.23-1.66]		1.14 [0.43- 3.05]	
Selling belongings	**5.85 [1.72-19.89]**		1.15 [0.42-3.17]		2.22 [ 0.79- 6.24]	
**Residence**						
Urban	1		1		1	
Rural	1.53 [0.81-2.88]	0.18	**2.51 1.26-4.97]**	0.007	**2.68 [1.33-5.41]**	0.006
***Clinical aspects***						
**Hemoptysis**						
No	1	0.21	1	0.14	1	0.05
Yes	0.69 [0.38- 1.23]		0.65 [0.36-1.16]		0.57 [0.31-1.02]	
***Knowledge, attitudes and beliefs***						
**Did not know how TB is transmitted**	**2.35 [1.26- 4.40]**	0.01	1.38 [0.75-2.54]	0.30	1.58 [0.86-2.91]	0.14
**Did not know what causes TB**	1.34 [0.66- 2.71]	0.41	**2.06 [1.02-4.13]**	0.03	1.30 [0.80-3.21]	0.16
**Did not know that TB treatment was free**	0.83 [0.48-1.41]	0.49	**0.47 [0.27-0.81]**	0.006	0.64 [0.37-1.10]	0.10
**Is there a link between AIDS and TB?**						
No	1	0.12	1	0.39	OR=1	0.41
Yes	0.57 [0.32-1.01]		0.74 [0.42-1.31]		0.79 [0.44-1.41]	
Did not know	0.60 [0.32-1.13]		0.65 [0.35-1.23]		0.65 [0.35-1.23]	
**What treatment can cure TB?**						
Self- medication	4.46 [0.47-42.67]	0.02	0.85 [0.09-8.30]	0.0003	1.89 [0.18-19.96]	0.0001
Medical care	1		1		1	
No answer	**2.37 [1.38- 4.05]**		**2.38 [ 1.37- 4.13]**		**3.05 [1.73- 5.40]**	
Nothing	4.22 [0.43-41.99]		5.80 [0.56-60.43]		5.24 [0.42-65.45]	
Traditional medicine	**2.20 [1.05-4.60]**		**5.70 [2.39-13.65]**		**5.02 [2.16-11.67]**	
***Access to and use of health services***						
**Referral by a health facility**						
Yes	1	0.04	1	0.18	1	0.63
No	**1.66 [1.02- 2.70]**		1.41 [0.86- 2.32]		1.13 [0.69-1.85]	
**Distance from home to closest service**						
≤ 1 km	1	0.01	1	0.09	1	0.15
1 to 5 km	1.25 [0.75- 2.07]		0.99[0.59-1.66]		1.24 [0.74- 2.08]	
≥ 5 km	**3.99 [1.52-10.48]**		**2.56[1.05-6.24]**		2.38 [0.99- 5.74]	
**First care received**						
Self-medication	1.58 [0.27-9.44]	0.10	0.77[0.14-4.15]	0.03	0.80 [0.15- 4.34]	0.09
Health center	1		1		1	
Hospital	0.92 [0.46-1.84]		**0.48[0.24-0.98]**		0.70 [0.35- 1.41]	
Pharmacist	**0.19 [0.04-0.99]**		0.50[0.13-1.98]		0.20 [0.04- 1.01]	
Private doctor	0.37 [0.13-1.03]		0.62[0.23-1.68]		0.72 [ 0.26-1.99]	
No health care	2.83 [0.31-26.29]		0.63[0.12-3.48]		0.62 [0.11- 3.40]	
Informal drug market	0.57 [0.28-1.17]		1.38[0.66-2.87]		0.95 [0.46-1.96]	
Traditional medicine	0.49 [0.21-1.14]		2.05[0.83-5.06]		2.46 [0.97- 6.24]	

***OR:****odds-ratio;****95% CI:****95% confidence interval.*

### Risk factors associated with an extended health-care system delay

In bivariate analysis, knowing that TB treatment is free and having received the primary care in a hospital were associated with a shorter HSD, while a low level of education, a low economic status, remote residence, living in a rural area, and the belief that traditional medicine can cure TB were associated with an extended HSD. In multivariate analysis, a low wealth score, having no knowledge about the correlation between AIDS and TB, a poor knowledge of TB treatment, and being treated at the HGRN were the three characteristics associated with an extended HSD.

### Factors associated with an extended total delay

Univariate analysis (Table 4), showed that having health insurance, unknown HIV serological status, knowing that TB treatment is free, and not knowing about the link between AIDS and TB were associated with a shorter TD. Living in a rural area, believing that traditional healing can cure TB and having started to undertake health care by using a traditional treatment appeared to be significantly associated with an extended TD. In multivariate regression analysis, a low economic status, the absence of hemoptysis, the belief in the efficacy of traditional and informal treatments, and being treated at either of Ndjamena’s hospitals were four characteristics associated with a longer TD.

**Table 4:** Comparison of the PD, HSD and TD with the findings in the literature

	**PD [Ref]**	**HSD [Ref]**	**TD [Ref]**
**African studies**	**2 to 7 days**[24,25]	**2 to 30 days**[16,26-31]	**26 to 44 days**[26,32]
	**14 days**[16,33-35]	**35 days**[36]	**52 to 62 days**[25,29,37]
	**21 to 60 days**[26-28,30,38-40]	**42 to 63 days**[2,24,25,38]	**77 to 120 days**[2,24,28,31,36,38,41,42]
**Our study**	**15 days**	**36 days**	**57.5 days**

***PD****: patient delay;****HSD****: health-care system delay;****TD****: total delay;****Ref****: reference.*

## Discussion

This study reveals a long delay in TB diagnosis, with an HSD 2.4 times longer than the PD (Table 1). The results show that a low economic status, a low level of education and the belief in the efficacy of traditional treatments were associated with extended diagnostic delays.

### Patient delay, health-care system delay and total delay

Lin X et al. found that TB infection spreads in the index case’s household after 30 days [3]. Three-fourths of the patients in this study began their TB treatment at least 33 days after the onset of symptoms (Table 1). Therefore, the delays in diagnosing TB observed in this study are likely to be important in the spread of this disease.

The median PD of 15 days is equal to the duration of a cough that should be considered suspicious for TB, according to the national program guidelines. The median HSD in this study is one of the longest observed, while the PD is one of the shortest compared to the findings in other settings (Table 5). This could be explained by the decision to include informal care in the definition of the primary care received by the patients in this study. Indeed, some authors consider the PD to be the time interval between the onset of symptoms and the first formal medical treatment received. Thus, the exclusion of informal and traditional health care from the definition of the primary care received seems to compound the patient’s role in the delay in TB diagnosis [6,7]. Therefore, the impact of informal care on the TD may be underestimated in resource-limited countries. For example, we observed that more than half of the patients visited a conventional care provider first and that those with formal care trajectories were likely to be diagnosed earlier. Therefore, traditional medicine and informal care should be considered part of the health-care system in studies conducted in developing countries. 

**Table 5:** Factors associated with delays exceeding their median value (multivariate analysis)

	**Extended patient delay**	**Extended health-care system delay**	**Extended total delay**
	**aOR [95% CI]**	**aOR [95% CI]**	**aOR [95% CI]]**
***Adjustment variables***			
**Gender**			
Male	1	1	1
Female	0.61 [0.35-1.07]	**1.67 [0.90-3.04]**	0.73 [0.41-1.30]
**Age groups (years)**			
15 to 24	1	1	1
25 to 34	0.77 [0.38-1.57]	0.57 [0.27-1.22]	0.53 [0.25-1.10]
35 to 44	1.03 [0.46-2.28]	1.13 [0.49-2.63]	0.62 [0.27-1.42]
45 to 55	0.90 [0.34-2.35]	0.47 [0.20-1.27]	0.59 [0.23-1.51]
55 and over	1.60 [0.45-5.58]	1.43 [0.40-5.06]	0.86 [0.28-2.91]
**Wealth score**			
1^st^ quartile (lowest)	**2.38 [1.08-5.25]**	**2.86 [1.30- 6.33]**	**3.75 [1.66-8.48]**
2^nd^	2.15 [0.97-4.76]	1.66 [0.74-3.70]	1.97 [0.90-4.44]
3^rd^	1.31 [0.62-2.79]	1.25 [0.59-2.67]	1.50 [0.70-3.24]
4^th^ (highest)	1	1	1
**Hospital**			
HM	1	1	1
HU	0.80 [0.35-1.81]	2.61 [1.07-6.36]	**2.78 [1.24-6.23]**
HGRN	1.04 [0.47-2.21]	**3.92 [1.83-8.42]**	**6.25 [2.96-13.22]**
***Selected variables***			
**Numbers of years of education**			**-**
0	0.88 [0.29-2.63]	**3.47 [1.01-11.88]**	
1 to 4	0.98 [0.31-3.10]	**4.71 [1.34-16.51]**	
5 to 6	0.42 [0.15-1.18]	2.89 [0.93-9.17]	
7 to 10	**0.33 [0.12-0.92]**	2.29 [0.76-6.95]	
≥ 10	1	1	
**What treatment can cure TB?**			
Medical care	1	1	1
No answer	**2.52 [1.40-4.50]**	**3.30 [1.71-6.35]**	**3.68 [1.71-7.92]**
Self-medication, traditional medicine, nothing	**2.15 [1.05-4.54]**	**5.46 [2.37-12.60]**	**3.76 [2.03-6.97**]
**Referral by a health facility**		**-**	**-**
Yes	**1**		
No	**1.75 [1.02- 3.02]**		
**Is there are link between AIDS and TB?**		1	**-**
No			
Yes	**-**	0.94 [0.48-1.84]	
No opinion		**0.37 [0.17- 0.80]**	
**Hemoptysis**			
Yes		-	**1**
No	-		**2.07 [1.06- 4.04]**

***aOR:****Adjusted odds-ratio;****95% CI:****95% confidence interval;****-:****unselected variable.*

### Determinants of patient delay

Several studies have shown that the inability to pay for health care is a barrier to seeking it [10-12]. Surprisingly, this was also the finding in this study, even though TB treatment is free. Indeed, patients bear certain direct and indirect costs (drugs, consultations, investigations, transportation, lost days of work, etc.) from the onset of symptoms to when TB is suspected. Although tests for TB are performed free of charge, patients still pay the rest of the expenses: food, transportation, lost income and so on. This prediagnostic cost can represent 7.1% of the median annual household income in Kenya, and patients may spend up to 125% of their monthly income to get a proper diagnosis in Ethiopia [13,14]. Mesfin et al found that spending time seeking care instead of earning money worsens TB patients’ financial burden and impoverishes their households [14]. This economic pressure may lead patients to delay their first visit to a doctor if the symptoms appear to be mild.

The PD seems to decrease when the level of education increases [15]. A higher level of education may be associated with a better knowledge of TB and a better understanding of the health-care system. Thus, more educated patients promptly consult a health professional shortly after the onset of symptoms. However, a higher level of education might also be associated with self-medication and the postponement of the first visit to a doctor.

Typically, patients with suspected TB would be seen in lower-level facilities and referred to the next level for further management. Thus, the referral system needs to be simple and efficient in order to reduce delays. When patients are not familiar with the referral system, they are likely to seek treatment outside the conventional services or make multiple visits to the same lower-level facilities without progressing upward. In our study, referral was associated with a shorter PD, which is contrary to the findings of other studies, where referral was associated with a longer PD (more obvious symptoms of TB due to a delayed first visit to a doctor) [16]. Surprisingly, there were few referrals in our study, despite the fact that the entire study population consisted of TB cases. This may be a reflection of the poor case-detection skills of lower-level health-care providers.

### Determinants of the health-care system delay

Similar to other studies which found that low income was associated with longer delays, we noted that low economic status lengthened the HSD [17,18]. Spending time seeking care and having to pay the necessary expenses to access it may impede the patient’s progression through the health-care system [19]. In Myanmar, Lönnroth et al showed that implementing measures to address the financial burden of TB can significantly shorten diagnostic delays [20]. Economic impediments to accessing health care are likely to contribute to the lengthening of the HSD in Chad, despite the fact that TB treatment is free there.

The organization of health care and its quality may affect the HSD [19]. Indeed, the centralization of TB diagnosis requires a visit to a hospital for the sputum smear test and a chest radiograph. In this study, the longest HSDs were associated with having been diagnosed in Ndjamena. This could be explained by the fact that this is a larger city with more-substantial health-care facilities, with the result that there are a larger number of potential steps in the pathway of care. Storla et al. do, in fact, call attention to the harmful role of repeated visits at the same level of care as one of the mechanisms that can contribute to diagnostic delay in TB [7]. The hierarchical level of care might also increase the risk of lengthening the HSD, given that the patients diagnosed at the HGRN seem to have had a longer HSD.

A poor knowledge of TB may lead to a longer HSD [19]. Believing in the efficacy of informal care and especially of traditional medicine in curing TB was significantly associated with longer HSDs in this study. The literature shows similar findings in different contexts, such as Vietnam, Nepal and South Africa [21-23]. These patients may use traditional healers as gatekeepers to enter the health-care system. The ability of these healers to identify TB symptoms and to then promptly refer the patient to a trained health professional could impact the HSD. Thus, training traditional healers on and involving them in the TB detection strategy might reduce the HSD.

### Determinants of total delay

The centralization of the point of diagnosis of TB, the referral pattern, the cost of care and the misunderstanding of the requirements of TB treatment influenced the TD in the same manner as they influenced the PD and the HSD. As a result, a longer TD was associated with a lower economic status, with the belief in the efficacy of informal treatment and with having been diagnosed at a Ndjamena hospital.

A low sensitivity of the TB screening criteria may also be a key factor in delays. Indeed, the TD was longer in the absence of hemoptysis. The inability of a health-care provider to suspect TB when the pulmonary signs are mild might explain this association [19]. This is probably one of the reasons why the TB detection rate remains so low in Chad.

Some factors seem to affect the first and the second phase of the pathway of care in opposite directions [19], and their effects on the TD may be the result of this opposite influence on the PD and the HSD. For example, being a woman was associated with a shorter PD, but paradoxically, it may be associated with a longer HSD. The women’s behaviour was unlikely to be significantly different from that of the men at the beginning of the trajectory of care, but afterwards, they were likely to encounter some gender-specific barriers once they entered the health-care system. The gender-specific parameters that may have been associated with the slow progression of women through the health-care system include a lack of financial independence, a lower social status, family responsibilities and a lack of respect from health-care providers*.* Consequently, public health interventions should be tailored to different circumstances*.*

### Limitations

Since it excluded patients who died before reaching the hospital and those who were too ill to be interviewed, this study may underestimate TB diagnostic delays in Chad. This should be taken into account when interpreting the results of this study. These results concern patients who had access to public tertiary hospitals in Ndjamena and Moundou**.** Since WHO estimated the TB case-detection rate at 26% in Chad in 2009 [8], there is a need to understand the behavior of patients who are not detected. Another study should help identify the determinants of their health care trajectories.

The multicenter design of this study enabled us to investigate the factors associated with the delayed initiation of TB treatment at two different levels of the health-care system and in two different cities and regions.

## Conclusion

The TD in Ndjamena and Moundou is too long. A fourth of the patients began their TB treatment at least 95 days after the onset of symptoms. The 286 patients in this study may have exposed 1740 members of their respective households to a risk of TB infection when they were infectious. The ability to pay for care, the level of education, knowledge of TB and knowledge of the organization of health care may determine the length of the delay in the diagnosis of TB. Significant differences in diagnostic delays might also depend on the quality of care, on the ability of health professionals to use the TB detection protocol, and on how they interact with the patients.

Implementing measures to inform the general public about TB and the availability of free TB treatment could help shorten diagnostic delays. Certain measures, such as microfinance, might improve the performance of the referral pattern by reducing the financial burden of TB for patients. Transporting sputum specimens from first-level facilities to the nearest hospitals could decentralize TB diagnosis without decreasing the quality of the sputum smear test. This decentralization would also reduce the cost incurred by patients to get diagnosed.

Training health workers on the management of TB via regular mentoring and supervision could improve the management of TB. The need to limit the transmission of the bacillus may encourage active screening of the households of contagious patients, despite the cost of this measure. Involving traditional healers and informal health professionals in the screening strategy might also facilitate patient access to TB diagnosis. Lastly, regular monitoring, a TB control program and the evaluation of this program are necessary to facilitate the use of public TB services.

## Competing interests

The authors declare no conflict of interest.

## Authors’ contribution

NNN designed the study protocol, collected and analysed the data and drafted the article. DN and NR revised the study protocol, collected the data and revised the article. MNN revised the study protocol and the article. MGS revised the article. VHF and PC revised the study protocol, supervised the data analysis and revised the article. All authors approve this submitted version of the article.

## Pre-publication history

The pre-publication history for this paper can be accessed here:

http://www.biomedcentral.com/1471-2458/12/513/prepub
